# Human Milk Oligosaccharides in Maternal Serum Respond to Oral Glucose Load and Are Associated with Insulin Sensitivity

**DOI:** 10.3390/nu15184042

**Published:** 2023-09-18

**Authors:** Marie-Therese Weiser-Fuchs, Elena Maggauer, Mireille N. M. van Poppel, Bence Csapo, Gernot Desoye, Harald C. Köfeler, Andrea Groselj-Strele, Slave Trajanoski, Herbert Fluhr, Barbara Obermayer-Pietsch, Evelyn Jantscher-Krenn

**Affiliations:** 1 Department of Obstetrics and Gynecology, Medical University of Graz, 8036 Graz, Austria; marie.fuchs@medunigraz.at (M.-T.W.-F.); gernot.desoye@medunigraz.at (G.D.); evelyn.jantscher-krenn@medunigraz.at (E.J.-K.); 2 Research Unit Early Life Determinants (ELiD), Medical University of Graz, 8036 Graz, Austria; 3 Institute of Human Movement Science, Sport and Health, University of Graz, 8010 Graz, Austria; mireille.van-poppel@uni-graz.at; 4 BioTechMed, 8010 Graz, Austria; harald.koefeler@medunigraz.at; 5 Core Facility Mass Spectrometry, Center for Medical Research, Medical University of Graz, 8036 Graz, Austria; 6 Core Facility Computational Bioanalytics, Center for Medical Research, Medical University of Graz, 8036 Graz, Austria; andrea.groselj-strele@medunigraz.at (A.G.-S.); slave.trajanoski@medunigraz.at (S.T.); 7 Department of Internal Medicine, Division of Endocrinology and Diabetology, Medical University of Graz, 8036 Graz, Austria; barbara.obermayer@medunigraz.at; 8 Department of Obstetrics and Gynecology, Endocrinology Lab Platform, 8036 Graz, Austria

**Keywords:** human milk oligosaccharides, pregnancy, oral glucose tolerance test, gestational diabetes mellitus, 3′-sialyllactose, hexosamine biosynthetic pathway, sialic acid, insulin sensitivity

## Abstract

(1) Background: Pregnancy presents a challenge to maternal glucose homeostasis; suboptimal adaptations can lead to gestational diabetes mellitus (GDM). Human milk oligosaccharides (HMOs) circulate in maternal blood in pregnancy and are altered with GDM, suggesting influence of glucose homeostasis on HMOs. We thus assessed the HMO response to glucose load during an oral glucose tolerance test (OGTT) and investigated HMO associations with glucose tolerance/insulin sensitivity in healthy pregnant women. (2) Methods: Serum of 99 women, collected at 0 h, 1 h and 2 h during a 75 g OGTT at 24–28 gestational weeks was analyzed for HMOs (2′FL, 3′SLN, LDFT, 3′SL) by HPLC; plasma glucose, insulin and C-peptide were analyzed by standard biochemistry methods. (3) Results: Serum 3′SL concentrations significantly increased from fasting to 1 h after glucose load, while concentrations of the other HMOs were unaltered. Higher 3′SL at all OGTT time points was associated with a generally more diabetogenic profile, with higher hepatic insulin resistance (HOMA-IR), lower insulin sensitivity (Matsuda index) and higher insulin secretion (C-peptide index 1). (4) Conclusions: Rapid increase in serum 3′SL post-oral glucose load (fasted-fed transition) indicates utilization of plasma glucose, potentially for sialylation of lactose. Associations of sialylated HMOs with a more diabetogenic profile suggest sustained adaptations to impaired glucose homeostasis in pregnancy. Underlying mechanisms or potential consequences of observed HMO changes remain to be elucidated.

## 1. Introduction

During pregnancy, the maternal body undergoes substantial transformations in virtually all organ systems such as the cardiovascular, immune, or metabolic system. These maternal adaptions are orchestrated by a complex interplay of several pregnancy-induced hormones, extracellular vesicles and other factors, and are pivotal to ultimately ensure proper fetal nutrition and development. This is especially true for glucose metabolism as regulation of glucose homeostasis substantially changes during gestation to warrant continuous glucose and energy supply to the fetus [1,2]. To ensure glucose flux along the concentration gradient from maternal to fetal circulation in mid to late pregnancy, maternal insulin sensitivity decreases, and insulin secretion increases [3] to compensate for the decreased insulin sensitivity. 

Maladaptation of gestational glucose metabolism leads to one of the most common pregnancy morbidities, gestational diabetes mellitus (GDM). GDM is referred to as spontaneous hyperglycemia developing during gestation. For decades, the oral glucose tolerance test (OGTT) has been a well-proven and widely used tool to detect GDM. Identification of GDM is of special importance since maternal and perinatal outcomes of (untreated) GDM may include severe morbidities [4]. GDM screening is performed in mid-pregnancy (24–28 weeks of gestation), and women must be fasted. Elevated glucose levels require further diagnostics and subsequent management of GDM.

Human milk oligosaccharides (HMOs) can be regarded as novel pregnancy-induced factors with potential roles in fine-tuning maternal adaptations. They are a group of highly bioactive glycans abundant in human milk, which have recently been found in low concentrations in the maternal circulation as early as the first trimester [5]. Serum HMO concentrations increase over the course of gestation, and their composition also changes. Oligosaccharides structurally identical to HMOs have also been found in cord blood [6] and amniotic fluid [7] suggesting that they can also reach the intrauterine compartment. HMO biosynthesis starts with lactose production facilitated by the lactose synthetase enzyme complex in the Golgi apparatus in differentiated lactocytes [8]. The lactose core can be further fucosylated or sialylated by specific enzymes to form the simplest HMOs fucosyllactose or sialyllactose, respectively. 2′-fucosyllactose (2′FL) and 3′-sialyllactose (3′SL) are the most abundant HMOs in pregnant serum, and the 2′FL/3′SL ratio increases with gestational age [5]. Lactose can also be elongated by galactose/N-acetylglucosamine disaccharide units leading to longer backbone structures which can be further modified by fucosyl- and/or sialyltransferases generating a pool of more than 200 known different HMO structures in human milk [9]. However, in serum, due to their much lower concentrations, profiles are less complex and also comprise oligosaccharides not commonly referred to as HMOs, such as 3′-sialyllactosamine (3′SLN) [5]. 3′SLN features a lactosamine instead of a lactose core, and is usually not reported in human milk. One decisive factor influencing HMO variation in pregnant and lactating women is their genetically determined secretor status. The secretor status depends on the activity of the secretor gene (*Se*) encoding for a specific fucosyltransferase, α-1,2-fucosyltransferase (FUT2) expressed in secretory tissues. Women with positive secretor status have α-1,2-fucosylated HMOs such as 2′FL, while secretor-negative women lack these specific fucosylations in their profile [9]. 

HMO structures determine their specific functions. In the context of the breastfed infant, many HMOs effects such as probiotic [10,11] or anti-infective effects [12] have been studied. Recent work also suggests systemic effects on immune modulation and metabolism [13,14,15]. However, the roles of serum HMOs during pregnancy on the maternal/fetal/placental unit remain elusive [6,16]. Whether and how serum HMOs are modulated by maternal factors such as diet/nutrient uptake, or whether HMO production is sensitive to transient plasma glucose fluctuations or affected by glucose dyshomeostasis, is not understood. We have recently shown that certain sialylated HMOs, namely 3′SL and 3′-sialyllactosamine (3′SLN), were altered in cord blood after pregnancies complicated by GDM suggesting that HMOs can be modulated by maternal glucose metabolism [17]. These same HMOs measured at early second trimester were found to precede maternal hyperglycemia and to be a strong predictor for the development of GDM in a population of overweight and obese women [18]. These findings suggest regulation of HMOs based on changes in glucose homeostasis during pregnancy, demanding further research. 

We thus here addressed two questions: (1) Can serum HMO concentrations be altered by a glucose load upon fasting suggesting a rapid glucose sensing/utilization? (2) Are HMOs associated with insulin sensitivity with potential consequences on pregnancy induced adaptations to altered glucose homeostasis? The OGTT used for GDM screening allows interrogation of these questions as it represents a standardized procedure to raise glucose levels after fasting and provides insights into glucose (patho)physiology in response to a glucose challenge. We hypothesized that HMO concentrations are modulated by changes in circulating glucose concentrations in mid-pregnancy in healthy pregnant women. To this end, we quantified HMO concentrations during the course of the OGTT and associated them with glucometabolic parameters such as insulin resistance and secretion. 

## 2. Materials and Methods

### 2.1. Study Design

Subjects (n = 99) selected for this study were participants of the HMO study, a prospective, observational, longitudinal study in healthy pregnant women (Clinical trial.gov #NCT05496712). The participants were recruited at the Department of Obstetrics and Gynecology of the Medical University of Graz between 2014 and 2019. Women came to the outpatient clinics for their routine care at four time points during pregnancy (10–14 weeks, 20–24 weeks, 24–28 weeks, and 33–35 weeks) and for delivery. A standard care oral glucose tolerance test was conducted at the clinics at mid-pregnancy (24–28 weeks of gestation).

Inclusion criteria for this study were an ongoing pregnancy at 10th–14th gestational week and a given written informed consent. Exclusion criteria included not wanting to give birth at the university hospital, gestational age above 14 weeks, multiple pregnancy, three or more consecutive miscarriages, an increased risk (≥1:100) after the combined test for chromosomal anomalies [19], fetal anomalies which are associated with possible growth or genetic anomalies, smoking, pre-pregnancy diabetes, maternal metabolic risk factors such as autoimmune conditions and increased risk for thromboembolic events needing anticoagulative therapy, as well as pre-pregnancy hypertension. The study complied with the Declaration of Helsinki guidelines as revised in the year 2000 and was approved by the ethical committee of the Medical University of Graz under the number EK# 26-380 ex13/14.

### 2.2. Anthropometrics

Height and body weight were measured using a fixed stadiometer and a digital scale, respectively, with light clothing and without shoes at all three time points. BMI was calculated as weight (kg)/height (m)^2^. Pre-pregnancy BMI was calculated based on self-reported pre-pregnancy weight. Maternal subcutaneous adipose tissue (SAT) thickness was assessed at each visit using a Lipometer, a patented optical device (EU Pat. Nr. 0516251) measuring back scattered light intensities corresponding to SAT [20]. Measurements were performed in 15 well-defined body sites in triplicates on the left side of the body. SAT thickness (in mm) of all sites was summed as estimation of total SAT.

### 2.3. Oral Glucose Tolerance Test (OGTT)

Study subjects underwent a 2 h 75 g OGTT screening for GDM in mid-pregnancy, between the 24th and 28th gestational week. They came to the clinics for this test in a fasted state (at least eight hours since the last meal). Venous blood samples were drawn at the fasted state, 1 h and 2 h after standardized oral uptake of 75 g glucose. Glucose threshold levels to meet the GDM criteria according to the IADPSG/WHO 2013 criteria were fasting plasma glucose ≥92 mg/dL (≥5.1 mmol/L), 1 h plasma glucose ≥180 mg/dL (≥10.0 mmol/L) or 2 h plasma glucose ≥153 mg/dL (≥8.5 mmol/L).

### 2.4. Human Milk Oligosaccharides Standards

2′-Fucosyllactose (2′FL), 3-fucosyllactose (3FL), lacto-N-tetraose (LNT), lacto-N-neotetraose (LNnT), lacto-N-fucopentaose 1, 2, and 3 (LNFP 1, 2, and 3), lacto-N-difucohexaose 1 (LNDFH1), and lacto-N-hexaose (LNH) were purchased from Dextra Laboratories, Reading, United Kingdom. Lactodifucotetraose (LDFT), 3′-sialyllactose (3′SL), 6′-sialyllactose (6′SL), 3′sialyllactosamine (3′SLN), 6′-sialyllactosamine (6′SLN), and sialyl-lacto-N-tetraose (LST) a, b, c and disialyllacto-N-tetraose (DSLNT) (Glycoset II) were purchased from Prozyme, Hayward CA. Linear B6 trisaccharide (Dextra Laboratories, Reading, UK) was used as internal standard.

### 2.5. HMO Isolation and Analysis by HPLC

Serum samples were prepared for later HMO analysis via high-performance liquid chromatography (HPLC) as previously published [5]. In brief, the internal standard (linear B6 trisaccharide, Dextra Laboratories) was added to diluted serum samples subjected to a chloroform/methanol extraction. The aqueous phase containing HMOs was further purified using solid phase extraction (SPE) with C18 columns (HyperSep C18 columns, Thermo Fisher Scientific, Waltham, MA, USA) followed by carbograph columns (Hypercarb columns, Thermo Fisher Scientific) in order to remove peptides and salts. The isolated and dried HMOs were then fluorescently labelled with 2-AB (2-aminobenzamide), and excess 2-AB removed with silica columns (HyperSep Silica columns, Thermo Fisher Scientific). The 2-AB-HMOs were separated by HPLC with fluorescence detection on a TSKgel Amide-80 column (Tosoh Bioscience, Tokyo, Japan). Retention times of HMO standards were used to annotate HPLC peaks. The amount of each individual HMO was calculated based on normalization to internal standard. 

### 2.6. Analysis of Glucometabolic Parameters 

Serum concentrations of insulin and C-peptide were measured using an ADVIA Centaur^®^ System (Siemens Healthcare, Erlangen, Germany). Concentrations are given as mg/dL for glucose, mU/mL for insulin, ng/mL for C-peptide. The analyses were carried out at the Department of Clinical Endocrinology of the Medical University of Graz.

Based on the measured metabolic parameters, a multitude of indices were calculated. Insulin resistance was estimated by the homeostasis model assessment insulin resistance (HOMA-IR) index. Further estimation of insulin sensitivity was obtained by calculating the Matsuda Index. β-cell function was estimated by calculating the HOMA-β index. To account for the role of the C-peptide, certain C-peptide-specific indices were calculated (C-peptide index 1 and 2); additionally, the formulae of the Matsuda Index and the HOMA-IR were adapted to fit in C-peptide levels. Insulin clearance (IC) was estimated by calculating the ratio of fasting C-peptide and fasting insulin (IC fasting) and via the ratio of the respective AUCs (IC AUC). Additionally, the insulinogenic and C-peptidogenic indices were calculated, ultimately leading to the calculation of the oral disposition indices (ODI) for insulin and C-peptide. A detailed description of the corresponding mathematical formulas can be taken from Supplementary Table S1. 

### 2.7. Statistics

Statistical analyses were performed using GraphPad Prism (version 9.1.2.; GraphPad Software, La Jolla, CA, USA) and SPSS (version 27; IBM SPSS, Chicago, IL, USA). Data are presented as means ± standard deviation. The variables were tested for normality using the Kolmogorov–Smirnov test.

HMO concentrations are presented as medians (interquartile range, IQR) in nmol/mL (fasting, e.g., 0 h, 1 h and 2 h). The area under the curve (AUC) for the time span between zero and one hour as well as between zero and two hours was calculated by assessing the difference between the concentrations of the respective time point by trapezoidal approximation. This type of calculation follows the trapezoid rule and offers a better description on the dynamics of the concentrations over the course of the OGTT.

Spearman correlation analyses were used to analyze associations among HMO concentrations, gestational age, body mass index (BMI) and maternal age. Differences of HMO concentrations over time were assessed by Friedman ANOVA followed by Dunn’s multiple comparison for differences between each time point. For all analyses, *p*-values of less than 0.05 were considered statistically significant.

Agglomerative hierarchical clustering with complete linkage on Euclidean distances based on 31 variables was performed in statistical program R version 4.2.2.

## 3. Results

### 3.1. Study Characteristics

Pregnant women in this cohort were, on average, 35 years old, and had a normal weight with an average pre-pregnancy BMI of 23.3 kg/m^2^. The mean BMI at delivery was 28.4 kg/m^2^, which corresponds to a mean BMI change of 5.0 kg/m^2^. The mean data of the maternal anthropometric parameters are listed in Table 1. 

Table 2 shows the maternal glucometabolic parameters, glucose, insulin and C-peptide, determined in the fasting state as well as one and two hours after the glucose load. The majority of the women included in the study had a normal glucose tolerance; five women were diagnosed with GDM according to the IADPSG criteria (5%). GDM cases were not different in basic characteristics. All cases were included in further analyses.

### 3.2. 3′SL Concentrations Increase during the OGTT

**Figure 1:**
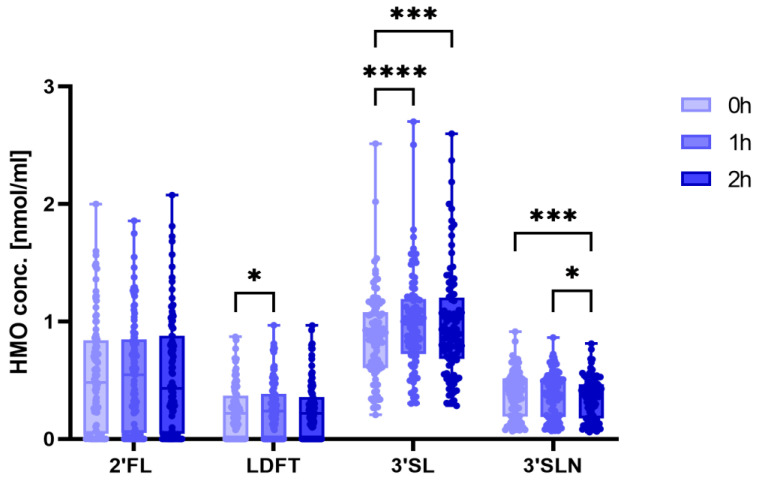
Human milk oligosaccharide concentrations in maternal serum change during a 75g-OGTT. 2′-Fucosyllactose (2′FL), lactodifucotetraose (LDFT), 3′-sialyllactosamine (3′SLN) and 3-sialyllactose (3′SL) concentrations are shown for each time point of the OGTT: 0 h, fasting state; 1 h, one-hour post-glucose load; 2 h, two hours post-glucose load. Significant differences between time points were determined using one-way ANOVA with Dunn‘s multiple comparison (**** *p* < 0.0001, *** *p* < 0.001, * *p* < 0.01). 3′SLconcentrations were significantly higher 1 h and 2 h post-load, 3′SLN concentrations was significantly lower 2 h post-load. The total study group was analyzed (n = 90).

HMO analysis in maternal serum during the OGTT identified 2′-fucosyllactose (2′FL), 3′-sialyllactosamine (3′SLN), lactodifucotetraose (LDFT) and 3′-sialyllactose (3′SL) as the four most abundant HMOs (Figure 1). We found the most prominent changes in 3′SL, as its median concentrations increased from fasting (0.89 nmol/mL; IQR 0.62–1.1 nmol/mL) to 1 h (1.00 mmol/mL; IQR 0.78–1.21 nmol/mL) and 2 h post-load (0.93 nmol/mL; IQR 0.71–1.24 mmol/mL) (*p* < 0.0001). 2′FL concentrations remained relatively stable with no significant changes, while LDFT concentrations increased slightly but significantly (*p* = 0.015) from baseline to one hour. 3′SLN concentrations decreased significantly from median fasting concentration of 0.42 nmol/mL (IQR 0.20–0.52 nmol/mL) to 0.39 nmol/mL (IQR 0.19–0.48) (*p* = 0.014) over the same time span. For all four HMOs, levels at the three time points (fasting, 1 h, and 2 h post-load) were highly correlated with each other. As expected, within the fucosylated HMOs, 2′FL and LDFT were highly positively correlated, and, to a lesser extent, within the sialylated HMOs, 3′SL and 3′SLN were correlated with each other. Twenty-six women (25%) showed an undetectable or very low 2′FL and were thus phenotyped as secretor-negative. When we analyzed secretor-positive women only, we found similar changes in HMOs over the course of OGTT.

### 3.3. Sialylated HMOs Are Associated with Glucose Tolerance and Insulin Sensitivity

**Figure 2:**
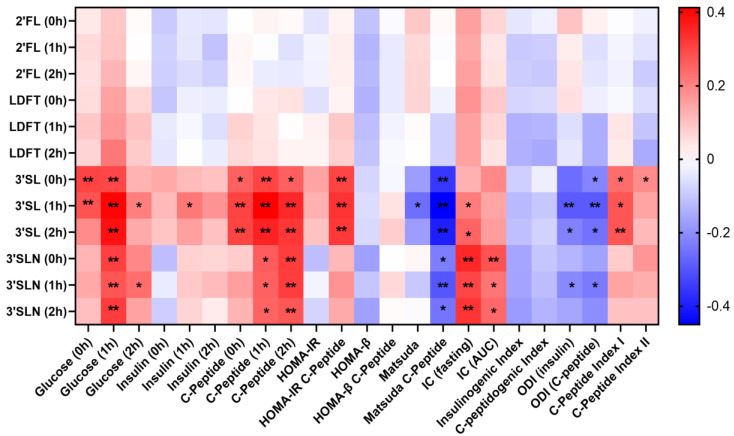
3′SL and 3′SLN are correlated with glucometabolic parameters. Heat map shows Spearman correlations between the four HMOs 2′FL, LDFT, 3′SL and 3′SLN and glucose, insulin and C-peptide (at 0 h, 1 h, 2 h post-glucose challenge during OGTT) and glucometabolic indices. The glucometabolic indices were calculated as described in Supplementary Table S1. Significant correlations are highlighted with asterisks (* *p* < 0.05, ** *p* < 0.01). The sialylated HMOs 3′SL and 3′SLN revealed numerous significant positive associations with glucose and C-peptide at several time points. Strong negative associations were found between 3′SL and Matsuda C-peptide as well as the oral disposition index (ODI). 3′SLN was also negatively associated with Matsuda C-peptide and showed additional strong positive associations with the insulin clearance (IC).

To assess the associations between HMOs and glucometabolic factors, Spearman correlation analyses were performed. The heatmap in Figure 2 shows the associations of the four most abundant HMOs found in maternal serum (2′FL, LDFT, 3′SL and 3′SLN) with the measured glucometabolic factors (glucose, insulin, C-peptide) as well as the calculated glucometabolic indices. The fucosylated HMOs 2′FL and LDFT were not significantly associated with glucometabolic factors/indices. The sialylated HMOs, 3′SL and 3′SLN, showed statistically significant positive associations with glucose and C-peptide at different time points. 3′SL at one-hour post-load (1 h) was highly significantly associated with glucose (r = 0.41, *p* < 0.01) and C-peptide (r = 0.39, *p* < 0.01) at the same time point. Fasting 3′SL (0 h) was positively associated with fasting C-peptide (r = 0.26, *p* < 0.05), 1 h C-peptide (r = 0.30, *p* < 0.01) and 2 h C-peptide (r = 0.26, *p* < 0.05). 3′SLN showed similar correlations as 3′SL, with most prominent positive correlations with 1 h glucose levels (0 h 3′SL: r = 0.31; 1 h 3′SL: r = 0.28; 2 h 3′SL: r = 0.32; *p* < 0.01). 3′SL at all three time points was, among others, strongly negatively associated with the Matsuda index (C-peptide), while 3′SLN was strongly positively associated with fasting insulin clearance (IC). When considering only the secretor-positive subgroup of the study cohort, we found additional negative associations between fasting 2′FL/LDFT and β-cell function as described by HOMA-*β* (C-peptide) (Supplementary Figure S1).

### 3.4. HMO Dynamics in Different Metabolic Profiles

**Figure 3:**
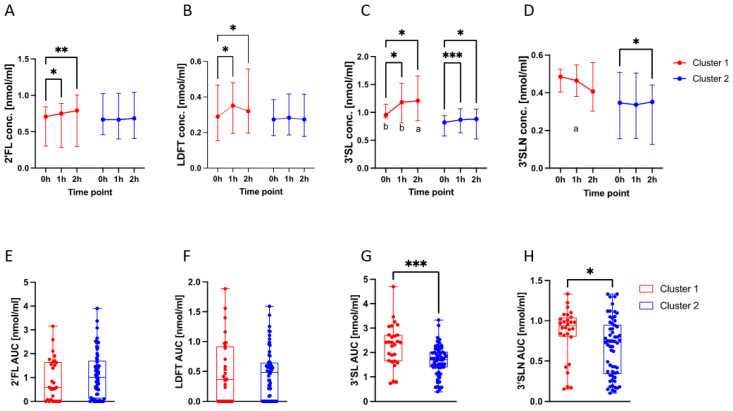
HMO dynamics during the OGTT differed among metabolic clusters. (**A**–**D**): HMO trajectories during OGTT in Clusters 1 (diabetogenic) and 2 (low risk) (median HMO concentration in nmol/mL with IQR). (**A**) 2′FL and (**B**) LDFT increased after 1 h, 2 h and from 1–2 h in Cluster 1 while remaining relatively stable in Cluster 2. (**C**) 3′SL concentration was significantly higher in Cluster 1 than in Cluster 2 at all time points (indicated by lower case letters a, b) and significantly increased in both clusters (indicated by asterisks). (**D**) 3′SLN concentration decreased 2 h post-load in Cluster 2 while slightly increasing in Cluster 2. 3′SLN concentration was significantly higher at 1 h in Cluster 1 (indicated by a). Significant differences between time points within the same cluster were determined using two-way ANOVA with Fisher‘s LSD testing (* *p* < 0.05; ** *p* < 0.005; *** *p* < 0.0005). Significant differences between Clusters 1 and 2 at the respective time points were determined using two-way ANOVA with Sidak’s multiple comparison (a = *p* < 0.05; b = *p* < 0.005); secretor-positive subcohort (n = 61). (**E**–**H**): Area under the curve (AUC) of the four HMOs 2′FL (**E**), LDFT (**F**), 3′SL (**G**) and 3′SLN (**H**) in the two metabolic clusters. Cluster 1 showed significantly higher 3′SL and 3′SLN AUC values. Significant differences were determined using Mann–Whitney Test (* *p* = 0.0381; *** *p* = 0.0002); total study cohort (n = 89).

Our finding that 3′SL significantly increased upon glucose challenge and was associated with glucometabolic traits raised the question whether HMOs, especially 3′SL, follows different trajectories during OGTT depending on the metabolic profile. To stratify the mostly healthy population of pregnant women, we performed cluster analysis. Hierarchical cluster analysis based on 31 glucometabolic variables showed the presence of two metabolic clusters (Supplementary Figure S2). Cluster 1 comprised 28, and Cluster 2 comprised 61 individuals from the study population. Cluster 1 was mostly characterized by significantly lower insulin sensitivity (Matsuda index; Matsuda C-peptide), higher insulin resistance (HOMA-IR C-peptide), higher (fasting) C-peptide levels, slightly elevated β-cell function (HOMA-β) and lower oral disposition index (ODI) than Cluster 2 (Supplementary Figure S3). Together with a higher BMI (25.5 kg/m^2^, IQR 23.4–28.7 kg/m^2^, vs. 21.4 kg/m^2^, IQR 19.7–22.9 kg/m^2^) and subcutaneous adipose tissue (SAT, 182 mm, IQR 158–202 mm vs. 126 mm; IQR 106–150 mm), as well as higher leptin levels, Cluster 1 showed a more diabetogenic metabolic profile with potentially higher risk for prediabetes or impaired glucose tolerance (IGT). This cluster also included the four GDM cases (one GDM case had missing values and was not included in the cluster analysis). We compared HMO trajectories during OGTT between the two clusters (Figure 3A–D). Median 2′FL concentrations were increased significantly after 1 h and from 1–2 h only in Cluster 1. LDFT concentrations in Cluster 1 increased after 1 h while being relatively stable in Cluster 2. 3′SL concentrations were higher in Cluster 1, with a significant increase after 1 h and 2 h. 3′SLN slightly decreased over time in both clusters. The 3′SL trajectory was found to be significantly different between the two clusters. Two-way ANOVA with Sidak’s multiple comparison found significant differences in 1 h and 2 h 3′SL concentrations between the clusters (*p* < 0.05) (Figure 3C).

We also compared the calculated area under the curve (AUC) of HMOs between 0 h and 2 h post-load between the two observed metabolic clusters (Figure 3E–G). The AUC estimates the total HMO concentrations throughout the whole two-hour OGTT. AUC (0 h to 2 h) of 3′SL and 3′SLN was significantly higher (*p* = 0.0002 and *p* = 0.0381, respectively) in Cluster 1 compared to Cluster 2 (Figure 3G,H). For the fucosylated HMOs, 2′FL and LDFT, no significant differences were found (Figure 3E,F). 

### 3.5. Correlations of 3′SL and 3′SLN with Glucometabolic Indices Differed per Cluster

We next investigated correlations between the sialylated HMOs, 3′SL and 3′SLN, and glucometabolic indices per cluster (Supplementary Figure S4). Spearman correlations revealed that 3′SL AUC was significantly correlated with the Matsuda C-peptide index (r = −0.61, *p* < 0.001), the HOMA C-peptide index (r = 0.44, *p* < 0.01) and trended to be significantly correlated with the oral disposition index (ODI) (r = −0.31, *p* = 0.056) in the more diabetogenic Cluster 1, while no significant correlations were found in Cluster 2 for these indices. In turn, 3′SL was positively correlated with insulin clearance in Cluster 2 (r = 0.35, *p* < 0.01), while there was no correlation in Cluster 1. 3′SLN was correlated with the Matsuda C-peptide (r = −0.34, *p* = 0.01) in Cluster 1. A highly significant positive correlation was found between 3′SLN and the insulin clearance in Cluster 2 (r = 0.47, *p* < 0.0001), with a similar trend in Cluster 1 (r = 0.26, *p* = 0.097). No significant correlation was found between 3′SLN and ODI. 

## 4. Discussion

We here describe two key findings: (1) Serum 3′SL changed upon glucose load over the course of an OGTT in healthy pregnant women. (2) HMO serum concentrations were associated with maternal glucometabolic profile, in particular lower insulin sensitivity associated with higher 3′SL concentrations at fasting and after glucose load.

### 4.1. Kinetics and Mechanisms of the HMO Changes Post Glucose Load

Among the HMOs investigated, only the sialylated HMO, 3′SL, highly significantly increased from fasting to post-glucose load. Remarkably, the observed 3′SL increase occurred relatively fast. One hour post-oral glucose intake, the median 3′SL concentration seemed to reach its peak (increased by 15%) before tending to decrease again at two hours post-load. However, we do not know the detailed kinetics of the HMO changes, whether they parallel the glucose increase which is known to reach the maximum at 30 min, or whether the 3′SL peak follows the glucose peak. Although less likely, we cannot exclude that the rise in serum 3′SL levels precedes the glucose peak. Thus, it would be interesting to investigate HMO trajectories over an OGTT with shorter intervals over a longer period of time, or in an euglycemic clamp experiment as well to monitor HMOs after a standardized meal. It might be interesting to see whether an increase in other nutrients and metabolites than glucose might modulate serum HMOs in a different way. Few studies so far have investigated changes in metabolites during an OGTT in pregnant women [21,22], and none have investigated HMOs. Most metabolites decrease upon glucose challenges such as acyl carnitines, amino acids and fatty acids [23]. Our findings that HMOs are affected by glucose fluctuation highlight the need to consider the fasting/fed status in future HMO studies. 

We can only speculate on the underlying mechanisms for the observed transient increases in 3′SL. Two possibilities may be considered: (1) de novo synthesis or (2) secretion of stored 3′SL. The most plausible scenario seems to be the de novo synthesis of 3′SL in the mammary gland upon serum glucose rise, despite the relative fast and subtle rise. It seems most likely that the plasma glucose peak precedes the 3′SL increase, and that higher glucose availability directly fuels HMO biosynthesis. In the mammary gland, glucose is taken up mostly by insulin-independent GLUT1 glucose transporters [24,25]. Glucose is a precursor for lactose, which is the prerequisite for HMO synthesis and can be further sialylated. Thus, 3′SL synthesis might either comprise the production of lactose, which is then sialylated, or the sialylation of the available lactose that might otherwise compete as a substrate for other glycosyltransferases. If plasma glucose changes mainly affected lactose production (without altering enzyme activities or availability of sialic acid or fucose), 3′SL and 2′FL would be expected to rise at the same rate. However, we found that the 3′SL as well as the 3′SL/2′FL ratios increased. This would fit with early studies in human lactation, showing that lactose biosynthesis is independent of fluctuations in plasma glucose [26]. Seemingly contradicting, we found that 3′SLN slightly decreased during the OGTT. However, this might be explained by potential differences in origin and regulation of 3′SLN compared to 3′SL, despite the common structural feature of sialylation. 3′SLN comprises lactosamine instead of lactose (it is not considered a ‘classical’ HMO found in human milk), and serum 3′SLN might stem from degradation of ubiquitous glycan conjugates. Our data might rather indicate that modification with sialic acid (SA) is increased upon glucose load in the mammary gland, either due to higher sialyltransferase activity or donor availability. Sialic acid can be produced by the body from glucose via the hexosamine biosynthetic (HBP) pathway leading to UDP-GlcNAc with a subsequent conversion of N-acetylmannosamine (ManNAc) in the SA biosynthesis pathway [27]. As this process is fast, an increase in SA might be a potential mechanism of increased glucose during the OGTT leading to increased sialylation. The HBP is highly responsive to glucose levels, and its flux is also significantly increased in diabetes [28]. Higher UDP-GlcNAc is also known to fuel sialic acid production and leads to increased sialylation of glycan conjugates a hallmark of many diseases such as cancer [29] and diabetes [30].

HMO concentrations in human milk have been shown to be associated with maternal diets [31] and to be affected by switching dietary carbohydrates (galactose vs. glucose) or energy sources (fat vs. carbohydrates) for a prolonged time [32]. These dietary effects on HMOs in milk seem to take time (days rather than hours) and may be mediated partly by effects on the microbiome, suggesting that in lactation, HMO production is rather stable and not responsive to fed or fasted state [33]. This might be contrary to pregnancy, when the mammary gland synthetic activity might be more sensitive to glucose homeostasis suggesting roles for HMOs as signal molecules in the maternal and fetal circulation.

### 4.2. Association of Sialylated Oligosaccharides with a Diabetogenic Metabolic Profile

In addition to the increased 3′SL upon exogenous glucose load, in our study, we found higher 3′SL and 3′SLN already at fasting conditions associated with a less favorable metabolic profile: higher glucose and C-peptide, lower insulin sensitivity. These associations were even more pronounced after glucose load. Furthermore, when we clustered the cohort according to their glucometabolic profile, we saw a significantly higher 3′SL AUC in the ‘unhealthier’ cluster presenting with a diabetogenic metabolic profile (higher BMI and subcutaneous adipose tissue, lower insulin sensitivity). These associations were different when we analyzed the two clusters separately, resulting in a loss of the association of 3′SL with insulin sensitivity in the metabolically healthier Cluster 2. On the other hand, Cluster 2 showed a positive association with insulin clearance. Together, this points towards a baseline increased 3′SL (and 3′SLN) associated with an underlying less favorable metabolic subtype in a mostly healthy cohort. This sustained increase in fasting sialylated oligosaccharides might also partly be due to an increased expression of respective glyco-synthetic enzymes, either lactose synthetase or α-2,3-sialyltransferase. Higher glucose availability was shown to increase lactose production partly by upregulation of β-1,4-galactosyltransferase (B1,4GalT) [34], part of the lactose synthetase complex in lactocytes. Similarly, high-glucose treatment of monocytes has been shown to induce upregulation of α-2,3-sialyltransferase 1 (ST3GAL1) [35], the sialyltransferase specifically required for 3′SL production in lactocytes. 

Higher sialylation has been shown in the context of many pathologies, mostly associated with inflammation. Increased serum SA is known to be a risk factor for cardiovascular diseases [36,37] and is increased in type 2 diabetes mellitus [38,39,40]. Non-pregnant subjects with impaired glucose tolerance (IGT) were shown to have higher total SA [41]. Pregnant women with GDM, but also with IGT, had higher levels of SA [42]. SA synthesis was shown to be stimulated in the liver and kidney under hyperglycemia [43], leading to higher sialylation at cell surfaces. This raises the interesting question of whether general sialylation (total SA) in serum is also associated with glucose metabolism in our cohort. 

Together, this association of elevated sialylation with an unfavorable immune–metabolic profile fits well with our findings that low insulin sensitivity is associated with higher concentrations of sialylated HMOs. Our findings in mostly healthy women are also well aligned with our previous finding in an overweight and obese cohort where serum 3′SL was associated with glucose metabolism and was found to be higher in women with GDM [6]. 

**Figure 4:**
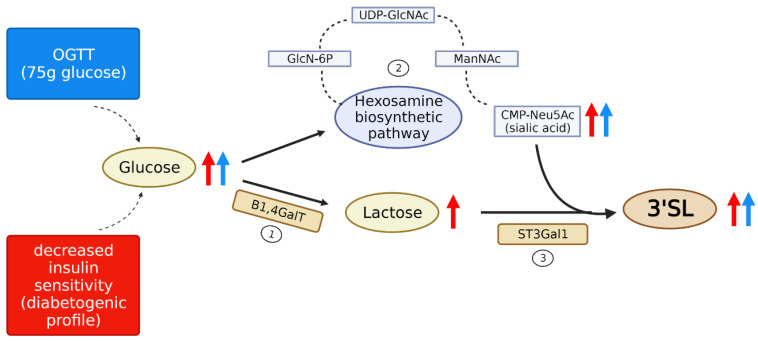
Proposed model of HMO regulation by glucose availability. According to our hypothesis, two scenarios contribute to the transient rise in 3′SL: Plasma glucose levels are elevated due to the 75 g glucose uptake in the OGTT (blue arrow), and individuals with generally decreased insulin sensitivity (diabetogenic profile) have subclinically increased basal glucose availability (red arrow). (1) Liu et al. showed that increased availability of glucose leads to an increased expression of β-1,4-galactosyltransferase (B1,4GalT), leading to the increased production of lactose [34]; however, as described by Neville et al., lactose production is independent of the short-term glucose availability in humans [26]. Considering these facts, we speculate that elevated lactose levels are predominantly observed in the case of decreased insulin sensitivity (red arrow), with elevated basal glucose levels. (2) Similarly, the hexosamine biosynthetic pathway (HBP) depends on glucose availability as described by Chari et al. [44]. Higher glucose availability leads to higher flux through the HBP, leading to higher CMP-NeuAc5 sialic acid donor substrate. (3) Ultimately, the sialyltransferase activity (ST3Gal1) was shown to be enhanced under high glucose concentrations [35] which could further contribute to the observed rise in 3′SL. The additive effect of altered insulin sensitivity through this hypothetic regulatory pathway would explain the observed differences in 3′SL between the clusters. The proposed pathways for HMO synthesis are located in the lactocytes of the mammary gland; however, HBP can also be activated in other peripheral tissues.

We here propose a model explaining the observed transient 3′SL increase during OGTT and the association of fasting 3′SL and 3′SLN levels with a generally more diabetogenic metabolic profile (low insulin sensitivity, higher fasting glucose) (Figure 4). Both findings, the 3′SL rise upon oral glucose load and the association of sialylated HMOs with lower insulin sensitivity in fasted and non-fasted status, seem to be influenced by elevated glucose availability to the mammary gland. In the former case, transient higher blood glucose is mediated by oral glucose intake upon fasting; in the latter case, it is rather due to an underlying subclinical glucose dysregulation (lower insulin sensitivity). Both of these might be fueling the hexosamine biosynthesis pathway to produce SA and build up 3′SL (and sialylated oligosaccharides). The consistently slightly increased plasma glucose availability might also upregulate enzymes involved in lactose and sialyllactose biosynthesis in the Golgi of lactocytes. 

### 4.3. Potential Roles for 3′SL as Signaling Molecules in Pregnancy

The finding that the higher 3’SL in serum associated with a more diabetogenic profile fits well with our previous findings in different cohorts, linking the higher 3’SL in serum with a low-grade inflammation setting, such as adiposity and insulin resistance [6] and pro-atherogenic profile (manuscript in preparation). However, the effects of 3′SL in the body are not known. Serum 3′SL is increased in pregnant compared to non-pregnant individuals, and concentrations seem to rise moderately over gestation [5]. In vivo and in vitro studies have attributed multiple effects to 3′SL, amongst them proangiogenic [17], anti-inflammatory and anti-atherogenic [45] effects, as well as effects on ganglioside biosynthesis in the brain. Based on our findings, we can only speculate on the consequence of the 3′SL increase, raising the question of whether 3′SL is protective or detrimental, counterbalancing or aggravating some of the pregnancy-induced unmet metabolic challenges. 3′SL in serum might be influencing a broad range of immunometabolic processes in blood and in target tissues as it interacts with galectins, siglecs and selectins. 3′SL has also been shown to alter glycan epitopes at the cell surface by changing the expression of sialyltransferases [46]. During pregnancy, excess glucose might be utilized for 3′SL production to serve as signal molecule, or provide SA as building block for ganglioside production in the fetus. By shunting glucose into the hexosamine pathway, it might be speculated that this can protect the fetus from exaggerated glucose flux across the concentration gradient, rather providing signals in the maternal and fetal compartment. 3′SL was shown to reach the fetal circulation [6], and thus might also influence placenta and fetus. An increased production of 3′SL under higher glucose availability might promote angiogenic effects in mother, fetus and placenta to adapt to the needs of a faster growing fetus and thus, higher oxygen and nutrients demand. In pregnancy, angiogenesis is important for maternal adaptations and development of placenta and fetus. The increase in 3′SL might also be protective to the maternal endothelial system provided that in vitro anti-inflammatory [47,48] and anti-atherogenic effects of 3′SL on macrophages [45] hold true in vivo. Attenuating maternal low-grade inflammation by 3′SL may also have metabolic effects ameliorating insulin sensitivity. It is known that sialylated glycans at the cell surface are involved in the regulation of cell–cell crosstalk, cell adhesion, or nutrient uptake such as glucose transport associated with insulin sensitivity/resistance. Moreover, components in glucose and insulin metabolism such as the insulin receptor are known to be heavily sialylated [49,50]. This might be increased with a general increase in sialylated epitopes associated with low-grade inflammation [51,52,53]. As sialylation and desialylation modulate the activity of the insulin receptor [54], increased abundance of sialylated HMOs and their postprandial upregulation could also modulate, directly or indirectly, insulin signaling. Interestingly, urinary 3′SL was proposed be a novel marker of unfavorable conditions in otherwise healthy individuals [55]. 

### 4.4. Strengths and Limitations

The study has several strengths. The cohort was prospectively established, and glucose metabolism was well characterized by multiple parameters. The availability of serum samples from each time point of a routinely performed OGTT made this possible and allowed us the investigation of the immediate effects of glucose load on HMO concentrations. 

The availability of C-peptide levels allowed for a robust assessment of insulin sensitivity as well as β-cell function. C-peptide is secreted in equimolar amounts to insulin as it is a by-product of insulin production in pancreatic β-cells. Stimulation by elevated glycemia lead to C-peptide and insulin release to portal circulation where they differ in half-life. While insulin has a half-life of only 3–5 min, C-peptide has a half-life of up to 30 min. In contrast to insulin, the C-peptide escapes first-pass hepatic metabolism, allowing the usage of the C-peptide as a more reliable marker of β-cell function [56]. This could explain why we found more significant associations with the C-peptide rather than with insulin. 

The strongest and most consistent associations were found with the Matsuda Index indicate; this index exhibits a stronger correlation during pregnancy with the euglycemic hyperinsulinemic clamp compared to other insulin sensitivity models (i.e., HOMA-IR) [57]. Substituting insulin by C-peptide when calculating the Matsuda Index has been validated during pregnancy [58], arguing for robustness of results in this study. 

We also have to acknowledge some limitations. Measurements of lactose and SA levels are lacking, which precluded testing our hypothesis on the mechanisms of increased 3′SL production via increased lactose and increased flux through the HBP. However, with the longitudinal data obtained during the OGTT, we were able to detect specific changes in HMOs that warrant further investigation with a special focus on HMO serum kinetics. Limiting the generalizability of our results is the fact that we had a mostly healthy, relatively small cohort. We did not have enough GDM cases to examine the differences in HMOs by GDM status. We thus performed cluster analyses based on glucometabolic parameters separating the cohort into two groups, one with a metabolically healthy profile and one with more diabetogenic profile. Larger cohorts allowing the analysis of more metabolic subtypes would be interesting. However, association of 3′SL with insulin sensitivity may help generate hypotheses that lead to a better understanding of the underlying mechanisms of GDM development. Implementation of HMO measurements with the measurements of glucose during an OGTT could be valuable, especially early in pregnancy. 

## 5. Conclusions

3′SL concentrations rapidly rise in response to a 75 g glucose load during an OGTT at 24–28 weeks of pregnancy in healthy women. We also found an association with higher-sialylated HMOs at baseline and AUC, especially 3′SL with a more diabetogenic metabolic profile in pregnancy, suggesting potential roles for 3′SL as biomarkers, but also modulators in glucometabolic health in pregnancy.

## Figures and Tables

**Table 1:** Basic characteristics of the study population.

Maternal Parameters	N	Mean ± SD
Age (years)	99	35.3 ± 4.2
Height (cm)	99	167.5 ± 6.7
Weight (kg, pre-pregnancy)	99	65.3 ± 12.5
BMI (kg/m^2^, pre-pregnancy)	99	23.3 ± 4.7
Weight (kg, delivery)	86	79.9 ± 13.2
BMI (kg/m^2^, delivery)	87	28.4 ± 4.8
Weight gain (kg)	84	14.2 ± 4.9
Parity (% nulliparous)	97	6.2

**Table 2:** Maternal glucometabolic parameters (means with standard deviation, SD).

Metabolic Parameters	N	Mean ± SD
Fasting Glucose (mg/dL)	98	77.7 ± 5.7
1 h Glucose (mg/dL)	97	119.5 ± 28.1
2 h Glucose (mg/dL)	95	105.6 ± 24.3
AUC ^1^ Glucose 0–2 h	94	209.0 ± 38.4
Fasting Insulin (mU/L)	98	8.2 ± 4.0
1 h Insulin (mU/L)	97	74.1 ± 44.1
2 h Insulin (mU/L)	96	56.4 ± 37.3
AUC ^1^ Insulin 0–2 h	94	101.2 ± 47.2
Fasting C-Peptide (ng/mL)	98	1.1 ± 0.4
1 h C-Peptide (ng/mL)	97	6.5 ± 2.3
2 h C-Peptide (ng/mL)	96	5.9 ± 2.3
AUC ^1^ C-Peptide 0–2 h	93	9.8 ± 3.0
**Glucometabolic Indices ^1^**	**N**	**Mean** **± SD**
HOMA-IR Index	97	1.6 ± 0.8
HOMA-IR Index (C-Peptide)	97	0.07 ± 0.03
HOMA-β	97	224.0 ± 127.1
Matsuda Index	93	7.3 ± 3.5
Matsuda Index (C-Peptide)	93	2819.9 ± 966.4
C-Peptide Index 1	97	1.5 ± 0.5
C-Peptide Index 2	93	11.0 ± 56.2
Insulin Clearance (fasting)	98	7.2 ± 2.0
Insulin Clearance (AUC)	93	0.11 ± 0.03
Insulinogenic Index	96	256.7 ± 246.3
C-Peptidogenic Index	96	1.11 ± 1.14
Oral Disposition Index (Insulin)	96	188.0 ± 184.9
Oral Disposition Index (C-Peptide)	96	16.8 ± 18.0

^1^ Area under the curve (AUC) was calculated by trapezoidal approximation of the respective concentrations during the OGTT. Glucometabolic indices were calculated according to the corresponding formulas listed in Supplemental Table S1.

## Data Availability

Not applicable.

## Supplementary Materials

The following supporting information can be downloaded at: https://www.mdpi.com/article/10.3390/nu15184042/s1. References [58,59,60,61,62,63] are cited in Supplementary file. Table S1: Mathematical formulas of the metabolic indices. Figure S1: Associations between HMOs and glucose metabolism in the secretor-positive subcohort. Figure S2: Hierarchical Cluster Analysis. Figure S3: Glucometabolic profiles of the two clusters. Figure S4: Correlations between 3′SL and 3′SLN and glucometabolic indices.

## Abbreviations

HMO	Human Milk Oligosaccharide
OGTT	Oral Glucose Tolerance Test
GDM	Gestational Diabetes Mellitus
HPLC	High-performance Liquid Chromatography
SPE	Solid Phase Extraction
2-AB	2-aminobenzamide
HOMA	Homeostasis Model Assessment
IC	Insulin Clearance
ODI	Oral Disposition Index
AUC	Area Under the Curve
2‘FL	2′Fucosyllactose
LDFT	Lactodifucotetraose
3′SL	3′Sialyllactose
3′SLN	3′Sialyl-N-Acetyllactosamine
SAT	Subcutaneous Adipose Tissue
BMI	Body Mass Index
IQR	Interquartile Range
SA	Sialic Acid
TSA	Total Sialic Acid
HBP	Hexosamine Biosynthetic Pathway
GlcNAc	N-Acetylglucsosamine
Neu5Ac	N-Acetylneuraminic Acid
B1,4GalT	β-1,4 Galactosyltransferase
ST3GAL1	Sialyltransferase 3
IGT	Impaired Glucose Tolerance
