# Supplementary material for: Human Milk Oligosaccharides in Maternal Serum Respond to Oral Glucose Load and Are Associated with Insulin Sensitivity
Source: Nutrients. 2023 Sep 18;15(18):4042. doi: 10.3390/nu15184042 (PMC10534497; doi:10.3390/nu15184042)
Supplement: Supplementary file 1 [file nutrients-15-04042-s001.zip › nutrients-2569896-supplementary.pdf]

Supplementary Materials

1. Mathematical formulas of the metabolic indices:

Supplementary Table S1: Overview on the calculated metabolic indices including the respective mathematical formulas.

Index	Formula
HOMA-IR [1]	$\frac{glucose\ 0h\ \left(\frac{mmol}{l}\right) * \ insulin\ 0h\ (\frac{mU}{ml})}{22,5}$
HOMA-IR (C-Peptide) [2]	$\frac{glucose\ 0h\ \left(\frac{mmol}{l}\right) * C - peptide\ 0h\ (\frac{nmol}{l})}{22,5}$
HOMA-β [3]	$20*(\frac{insulin\ 0h\ (\frac{mU}{ml})}{glucose\ 0h\ \left(\frac{mmol}{l}\right) - 3,5})$
HOMA-β-C-peptide [3]	$20*(\frac{C-peptide\ 0h\ (\frac{nmol}{l})}{glucose\ 0h\ \left(\frac{mmol}{l}\right) - 3,5})$
Matsuda Index [4]	$\frac{10.000}{\sqrt{Glc\ 0h\ \left(\frac{mg}{dl}\right) * Ins\ 0h\ (\frac{mU}{ml}) * MW(Glc)\left(\frac{mg}{dl}\right) * MW(Ins)(\frac{mU}{ml})}}$
Matsuda Index (C-Peptide) [5]	$\frac{500.000}{\sqrt{Glc\ 0h\ \left(\frac{mg}{dl}\right) * C - Pep\ 0h\ (\frac{ng}{ml}) * MW(Glc)\left(\frac{mg}{dl}\right) * MW(C - Pep)(\frac{ng}{ml})}}$
C-Peptide Index 1 (CPI) [3]	$100* \frac{C-Peptide\ 0h\ (\frac{ng}{ml})}{Glucose\ 0h\ (\frac{mg}{dl})}$
C-Peptide Index 2 (CPII) [3]	$100* \frac{C-Peptide\ 2h\ \left(\frac{ng}{ml}\right) - C-Peptide\ 0h\ (\frac{ng}{ml})}{Glucose\ 2h\ \left(\frac{mg}{dl}\right) - Glucose\ 0h\ (\frac{mg}{dl})}$
Insulin clearance (fasting) [6]	$C - Peptide_{0h}\ (\frac{nmol}{l}) \Big/ Insulin_{0h}\ (\frac{nmol}{l})$
Insulin clearance (AUC) [6]	$C - Peptide_{AUC}\ (\frac{nmol}{l}) \Big/ Insulin_{AUC}\ (\frac{nmol}{l})$
Insulinogenic Index [2]	$\frac{Insulin\ 1h - 0h\ (\frac{pmol}{l})}{Glucose\ 1h - 0h\ (\frac{mmol}{l})}$
C-Peptidogenic Index [2]	$\frac{C - Peptide\ 1h - 0h\ (\frac{nmol}{l})}{Glucose\ 1h - 0h\ (\frac{mmol}{l})}$
Oral Disposition Index (Insulin) [2]	$Insulinogenic\ Index \Big/ HOMA - IR$
Oral Disposition Index (C-Peptide) [2]	$C - Peptidogenic\ Index \Big/ HOMA - IR\ (C - Peptide)$

2. **Associations between HMOs and glucose metabolism in the secretor-positive subcohort**

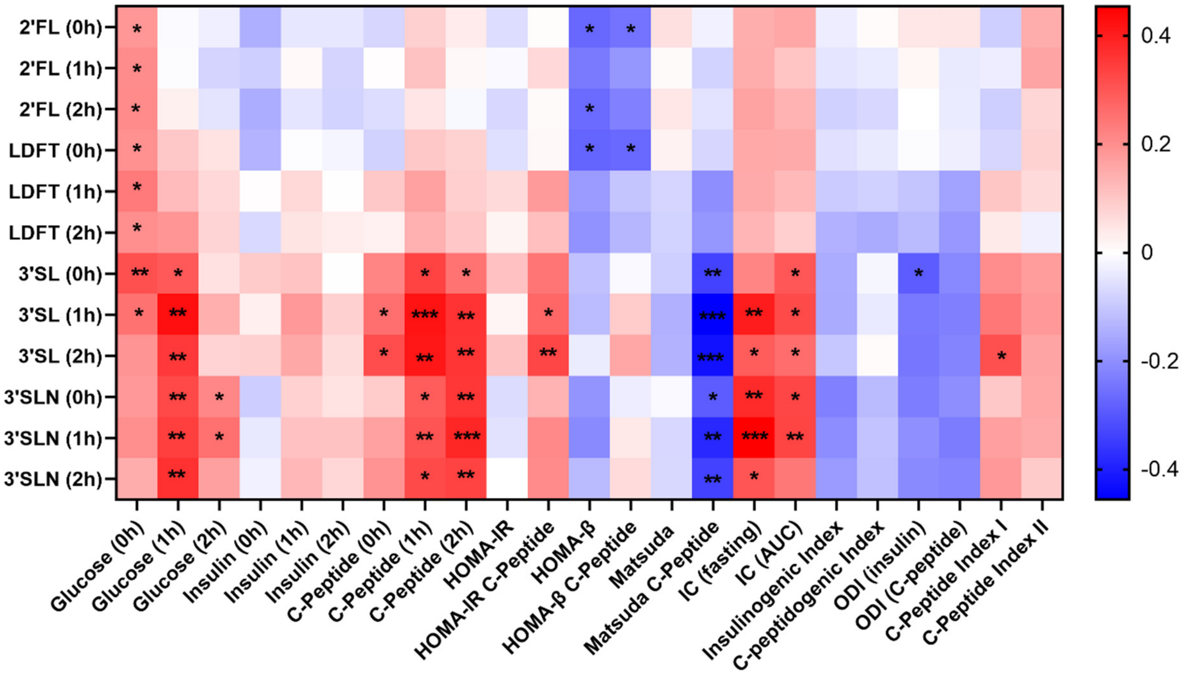

Supplementary Figure S1: HMOs are associated with glucometabolic parameters in the secretor-positive group of the study population. Heatmap shows spearman correlations between the four HMOs 2′FL, LDFT, 3′SL and 3′SLN and glucose, insulin and C-peptide (at 0h, 1h, 2h post glucose challenge during OGTT) and glucometabolic indices. Significant correlations are highlighted with asterisks (*$p<0.05$, **$p<0.01$). The sialylated HMOs 3′SL and 3′SLN showed numerous significant positive associations with glucose, C-peptide and numerous indices at several time points, which is similar to the results of the total study group. Additional positive associations were found between 2′FL and LDFT and fasting glucose levels in the secretor-positive fraction. Fasting 2′FL and LDFT were also found to be negatively associated with HOMA-β and HOMA-β C-Peptide in the secretor-positive subcohort.

3. Hierarchical cluster analysis

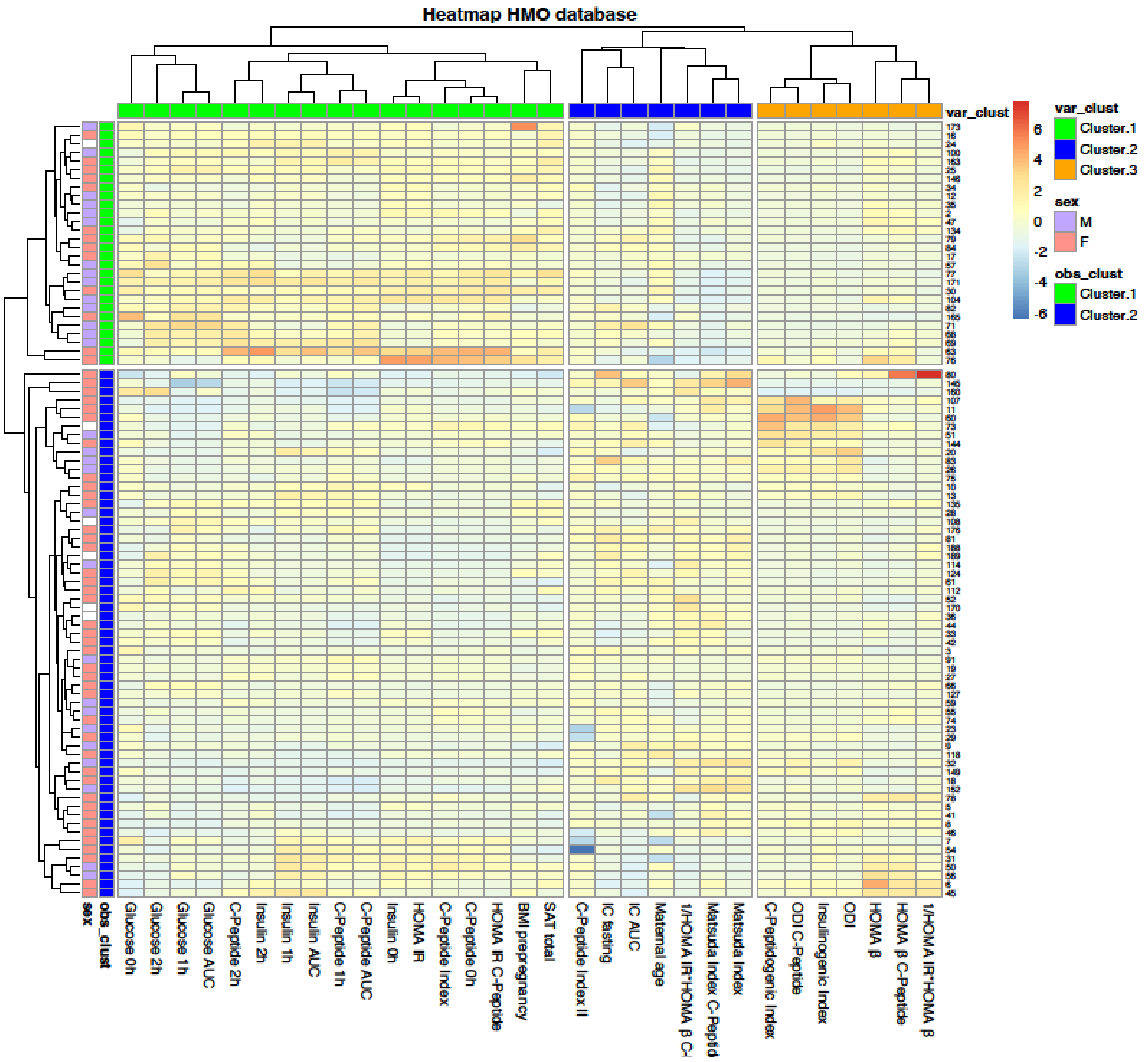

Supplementary Figure S2: Heat map of Hierarchical Clustering. Cluster analysis was performed based on 31 variables and showed the presence of two distinct clusters within the study group: Cluster 1 (n=28) and Cluster 2 (n=61).

4. Glucometabolic profiles of the two clusters

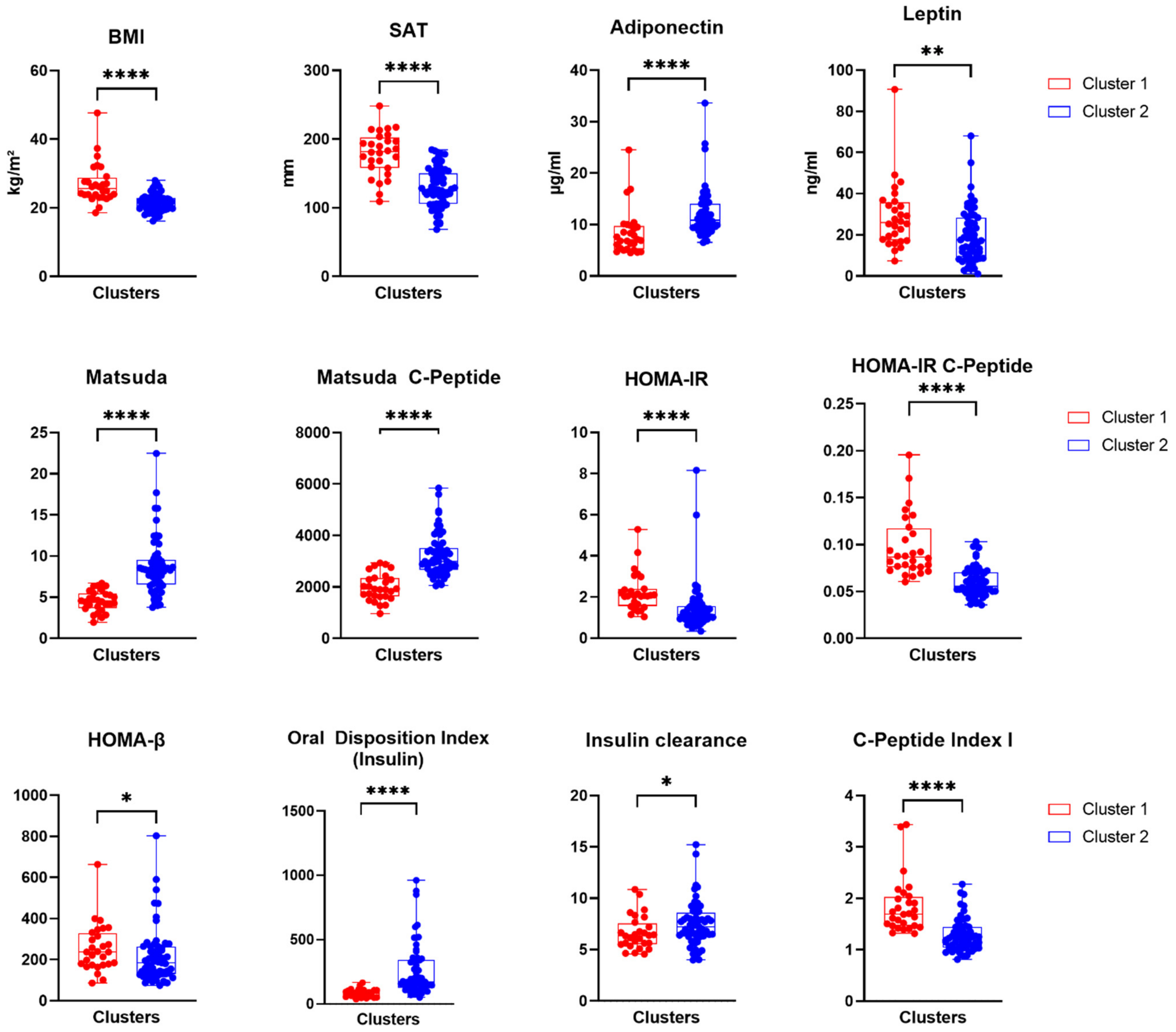

Supplementary Figure S3: Comparison of the glucometabolic profiles between cluster 1 and 2. BMI, body mass index (kg/m²); SAT, subcutaneous adipose tissue (mm). Significant differences are presented by asterisks (*p<0.05; **p<0.03; ****p<0.0001). Cluster 1 and 2 differ significantly in their glucometabolic profiles. BMI, SAT, leptin, HOMA-IR, HOMA-IR (C-Peptide), HOMA-β and C-Peptide Index I are higher in cluster 1. In contrast, cluster 2 showed higher adiponectin, Matsuda, Matsuda C-Peptide, oral disposition index (insulin) and insulin clearance. Significant differences between the clusters were determined using Multiple Mann-Whitney with FDR (Benjamini, Krieger and Yekutieli).

5. Correlations between 3’SL and 3’SLN and glucometabolic indices

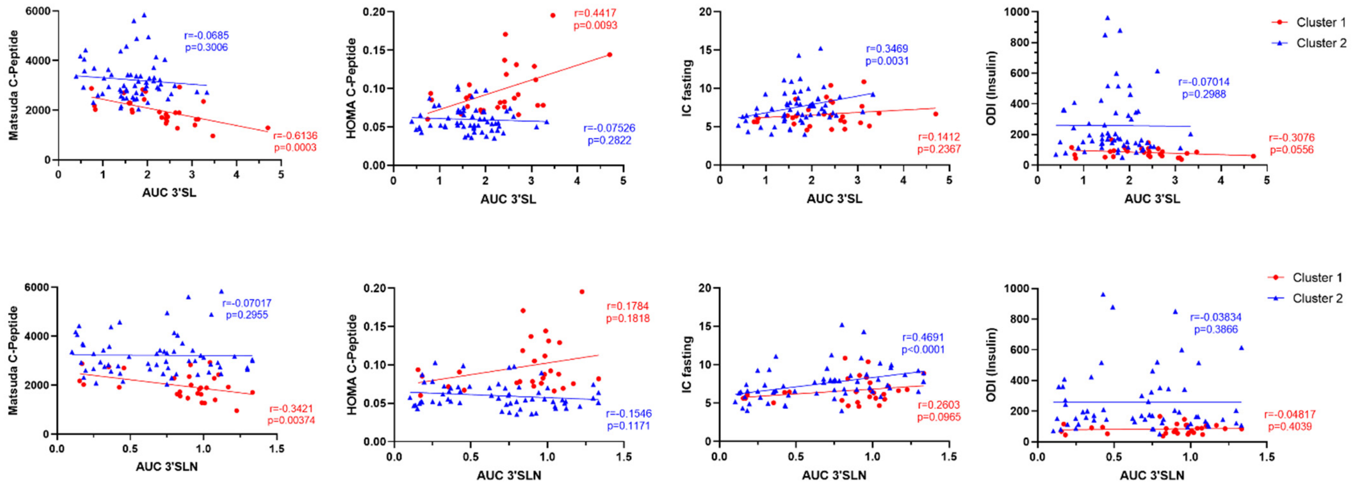

Supplementary Figure S4: Correlations of 3’SL and 3’SLN and glucometabolic indices was different in the two metabolic clusters. Area under the curve (AUC) of 3’SL and 3’SLN was used to estimate the correlations with glucometabolic indices throughout the 2h-OGTT. 3’SL is correlated with Matsuda C-Peptide, HOMA-C-Peptide index and ODI in cluster 1 and with fasting insulin clearance (IC) in cluster 2. 3’SLN is correlated with Matsuda C-Peptide in cluster 1 and with fasting IC in cluster 2. Correlations are Spearman correlations with the respective r and p values.

6. References

1. Rita S. Patarrãoa, Wilford Wayne Lautt b, Maria Paula Macedoc,d, *Assessment of methods and indexes of insulin sensitivity.* 2012.
2. Kim, J.D., et al., *C-Peptide-Based Index Is More Related to Incident Type 2 Diabetes in Non-Diabetic Subjects than Insulin-Based Index.* Endocrinol Metab (Seoul), 2016. **31**(2): p. 320-7.
3. Alvarez-Nava, F., et al., *Insulin Sensitivity and Pancreatic beta-Cell Function in Ecuadorian Women With Turner Syndrome.* Front Endocrinol (Lausanne), 2020. **11**: p. 482.
4. DeFronzo, R.A. and M. Matsuda, *Reduced time points to calculate the composite index.* Diabetes Care, 2010. **33**(7): p. e93.
5. Radaelli, T., et al., *Estimates of insulin sensitivity using glucose and C-Peptide from the hyperglycemia and adverse pregnancy outcome glucose tolerance test.* Diabetes Care, 2010. **33**(3): p. 490-4.
6. Semnani-Azad, Z., et al., *Determinants of longitudinal change in insulin clearance: the Prospective Metabolism and Islet Cell Evaluation cohort.* BMJ Open Diabetes Res Care, 2019. **7**(1): p. e000825.
